# Prostatitis-Related Male Infertility: From Inflammation and Dysbiosis to Sperm DNA Damage

**DOI:** 10.3390/diagnostics16050722

**Published:** 2026-02-28

**Authors:** Aris Kaltsas, Nikolaos Pantazis, Vasileios Tzikoulis, Christos Roidos, Natalia Palapela, Chara Tsiampali, Evangelos N. Symeonidis, Athanasios Zachariou, Nikolaos Sofikitis, Fotios Dimitriadis

**Affiliations:** 1 Third Department of Urology, Attikon University Hospital, School of Medicine, National and Kapodistrian University of Athens, 12462 Athens, Greece; ares-kaltsas@hotmail.com; 2 First Department of Urology, Faculty of Medicine, School of Health Sciences, Aristotle University of Thessaloniki, 54124 Thessaloniki, Greece; nikospant94@gmail.com (N.P.); bill1996tziko@gmail.com (V.T.); drchriroid22@gmail.com (C.R.); 3 Medical Faculty, Medical University of Sofia, 1431 Sofia, Bulgaria; nataliapalapela21@gmail.com; 4 Independent Researcher, 55131 Thessaloniki, Greece; x.tsiampali@gmail.com; 5 Department of Urology II, European Interbalkan Medical Center, 55535 Thessaloniki, Greece; evansimeonidis@gmail.com; 6 Department of Urology, Faculty of Medicine, School of Health Sciences, University of Ioannina, 45110 Ioannina, Greece; zahariou@otenet.gr (A.Z.); nsofikit@uoi.gr (N.S.)

**Keywords:** prostatitis, chronic prostatitis/chronic pelvic pain syndrome, male infertility, leukocytospermia, oxidative stress, sperm DNA fragmentation, seminal microbiome, interleukin-8, prostasomes, assisted reproduction

## Abstract

Prostatitis includes infectious and noninfectious inflammatory phenotypes that can impair male reproductive potential and may influence couple-level reproduction via seminal inflammatory and microbial exposure. This review summarizes mechanisms linking prostatic inflammation and dysbiosis to semen dysfunction and sperm DNA damage and proposes an infertility-oriented diagnostic and management framework. This is a narrative review of clinical and translational evidence addressing semen inflammation, oxidative stress, sperm DNA fragmentation (SDF), microbiome signatures, and reproductive outcomes in prostatitis (National Institutes of Health (NIH) categories I-IV). Across prostatitis phenotypes, leukocytospermia and elevated seminal cytokines (especially interleukin-8) are associated with impaired motility, altered viscosity and liquefaction, oxidative stress, and higher SDF. Persistent infection or dysbiosis may sustain immune activation and redox injury, while ductal remodeling and pain-related sexual dysfunction can further reduce natural conception. Seminal cytokines and microbes may affect female reproductive tract biology, although clinical outcome data remain limited. Prostatitis-related infertility requires evaluation beyond routine semen analysis. A biomarker-guided workup integrating inflammatory markers, oxidative stress testing, targeted microbiology (culture plus nucleic acid amplification tests when indicated), SDF testing in selected men, and imaging when obstruction is suspected can identify treatable drivers and guide timing and selection of assisted reproduction strategies. Future studies should standardize fertility endpoints and validate biomarker-guided and microbiome-directed interventions.

## 1. Introduction

Prostatitis is a common and clinically important urogenital condition that affects up to 10% of men, with peak incidence in the late thirties and forties [1,2]. It spans acute bacterial infection through chronic inflammation, and chronic prostatitis/chronic pelvic pain syndromes (CP/CPPS) account for most cases and often show no identifiable pathogen [1,2]. Infections and inflammatory disorders of the male genital tract contribute to an estimated 10–35% of male infertility, placing prostatitis at a key intersection between inflammation and reproductive dysfunction [3,4,5]. Men with chronic prostatic inflammation frequently have impaired semen quality, with reduced ejaculate volume, reduced motility, and abnormal morphology, together with leukocytospermia and enrichment of pro-inflammatory cytokines [3,4,6]. High seminal interleukin (IL)-8, a marker of prostate-derived inflammation, correlates inversely with sperm motility, while excess reactive oxygen species (ROS) generated by activated leukocytes produce oxidative stress that routine semen analysis does not capture [7,8]. Men with chronic pelvic pain syndromes show higher sperm DNA fragmentation (SDF) indices than controls, and dysregulation of the genitourinary microbiome, including asymptomatic infection and seminal dysbiosis, may further amplify inflammation, oxidative damage, and ductal fibrosis. Prostatitis is therefore both a clinically important and mechanistically complex contributor to male infertility [7,8]. Accordingly, this review focuses on translating these mechanistic pathways into an infertility-oriented diagnostic workup and management strategy, including clinically actionable decision points for assisted reproduction.

Despite these links, evaluation of prostatitis-related infertility remains fragmented [9,10]. Conventional workups often ignore subclinical prostatic inflammation. Many affected men have apparently normal semen parameters, while others show mild reductions in motility or subtle morphologic changes that are easily dismissed [9,10]. There is no widely adopted protocol that integrates markers of prostatitis into male infertility assessment, and tests for seminal cytokines, oxidative stress markers, or advanced microbiological screening are used irregularly [9,10]. Treatable inflammatory or infectious drivers of male subfertility may therefore remain unrecognized [11]. In this context, available evidence is synthesized to support a practical, biomarker-guided framework that links inflammatory, oxidative, microbial, and obstructive axes to targeted testing and stepwise treatment escalation.

A narrative literature review was conducted. PubMed/MEDLINE and Embase were searched from database inception through 31 December 2025 for English-language clinical and translational studies using combinations of keywords related to prostatitis (National Institutes of Health (NIH) categories I–IV; CP/CPPS), leukocytospermia, IL-8 and other cytokines, oxidative stress (ROS and oxidation–reduction potential (ORP)), sperm DNA fragmentation (SDF/DNA fragmentation index (DFI)), microbiome/dysbiosis, and infertility/assisted reproduction. Reference lists of relevant clinical practice guidelines and key review articles were additionally screened to identify further eligible studies, with priority given to clinical practice guidelines, systematic reviews/meta-analyses, and prospective clinical studies, complemented by mechanistic and epidemiological evidence to contextualize the proposed diagnostic framework.

## 2. Prostate Biology and Prostatitis Pathophysiology in Relation to Male Fertility

### 2.1. Prostatic Secretory Biology: Molecular Determinants of Semen Function

The prostate shapes seminal plasma by secreting zinc- and citrate-rich fluid and proteases (kallikreins, including prostate-specific antigen (PSA)) that help regulate semen pH and liquefaction, thereby supporting sperm transport and progressive motility [12,13,14].

It also releases extracellular vesicles (EVs, known as prostasomes) that interact with sperm after ejaculation and modulate motility, capacitation timing, and local immune/oxidative buffering within the ejaculate [15]. These baseline functions provide context for why prostatitis can present with abnormal viscosity/liquefaction and downstream inflammatory and oxidative signatures in semen.

### 2.2. Prostatitis Pathophysiology: Secretory Disruption and Fertility Signals

Prostatitis spans infectious and noninfectious inflammatory phenotypes (NIH categories I-IV) and can compromise fertility by disturbing prostatic secretions and establishing a seminal environment that is unfavorable for sperm [1]. Table 1 summarizes the NIH prostatitis categories and highlights fertility-relevant characteristics to support infertility-oriented phenotyping and workup.

From a fertility-focused standpoint, prostatitis is most usefully interpreted through a small set of clinically actionable domains: disruption of prostatic secretory function (manifesting as abnormal semen viscosity and/or delayed liquefaction with accompanying changes in prostatic biochemical markers); an increased inflammatory burden (including leukocytospermia and enrichment of prostate-derived cytokines); heightened oxidative stress with potential downstream sperm DNA damage; and—among selected patients—chronic tissue remodeling that may contribute to functional outflow impairment [6,14,16,17]. These domains are discussed mechanistically in Section 3 and then integrated into the stepwise, infertility-oriented diagnostic approach proposed in Section 5.

## 3. Mechanistic Pathways Linking Prostatitis to Sperm Dysfunction

### 3.1. Inflammation and Immune Dysregulation

Chronic prostatitis is associated with a sustained inflammatory milieu in the prostate and ejaculate that is not captured by leukocyte counts alone. Studies of seminal plasma cytokines consistently report higher IL-6, IL-8, and tumor necrosis factor alpha (TNF-α) in affected men, and these elevations are typically associated with poorer motility and morphology [18,19,20].

Innate immune sensing is a plausible upstream driver. Pattern-recognition receptors on prostatic epithelium and on the sperm can detect microbial motifs and danger signals and promote chemokine release and leukocyte recruitment. Experimental work summarized in recent reviews suggests that toll-like receptor (TLR) 2 and TLR4 activation on sperm is associated with reduced motility and viability and with altered capacitation signaling through calcium- and cyclic nucleotide-dependent pathways [21,22]. Complement activation and damage-associated molecular patterns (e.g., high-mobility group box 1) may further amplify inflammation and help sustain it after pathogen clearance in some settings [23,24].

Inflammasome signaling has also been implicated. NOD-, LRR- and pyrin domain-containing protein 3 (NLRP3) activation promotes maturation of IL-1β and IL-18 and can trigger inflammatory cell-death programs in epithelial cells. In translational studies, NLRP3 components are reported to be higher in expressed prostatic secretions from men with chronic pelvic pain syndromes than in controls, and in experimental models, NLRP3 inhibition is associated with reductions in inflammatory injury and pain-related behaviors [25,26,27,28]. Purinergic signaling via P2X7 may facilitate NEK7–NLRP3 interaction and act as a feed-forward amplifier in these models [27].

Neutrophil extracellular traps (NETs) provide an additional effector pathway. In inflammatory semen, leukocytes can extrude chromatin networks decorated with histones and granular enzymes that physically entangle sperm and are associated with reduced progressive motility and viability in experimental systems. NET components may also increase local oxidative stress and thereby contribute to membrane and DNA injury in nearby sperm [29,30].

Other inflammatory mediators may be relevant in specific phenotypes. IL-17- and IL-18-related pathways and prostanoid signaling have been linked to impaired motility and fertilization parameters in recent analyses, but the strength of clinical outcome data remains variable [21,23,31]. Antisperm antibodies occur in a minority of men and are rarely an isolated cause of infertility; assay heterogeneity and the ability of assisted reproduction to bypass antibody effects limit their standalone clinical weight [21,23,31].

Clinically, documenting an inflammatory semen profile (e.g., leukocytospermia and/or elevated IL-8) should prompt targeted microbiological assessment and phenotype-directed anti-inflammatory management rather than reliance on routine semen parameters alone [9]. Because inflammation and oxidative stress often co-occur, oxidative stress testing is reasonable in this context, and SDF testing can be considered in selected men to quantify downstream reproductive risk and to guide counselling around antioxidants and the timing or choice of assisted reproduction [10].

### 3.2. Oxidative Stress and Sperm DNA Integrity

Inflammation in the prostate and seminal tract is commonly accompanied by increased ROS generation in semen, largely from activated leukocytes. When oxidant production exceeds seminal antioxidant capacity, a shift toward oxidative stress is observed, and this state is consistently associated with reduced motility and vitality and with impaired sperm function in clinical and experimental studies [6,7,9]. Leukocytospermia is frequently the proximate clinical marker of this redox imbalance [6,7,9].

Lipid peroxidation is a key lesion because sperm membranes are rich in polyunsaturated fatty acids. Reactive aldehydes such as 4-hydroxynonenal can form adducts with axonemal proteins and disturb ion-channel function, which may compromise hyperactivation and progressive motion. Human studies also implicate oxidized lipid mediators (including neuroprostanes) and redox-driven post-translational protein modifications in altered motility and capacitation timing [31,32].

Nuclear DNA is particularly vulnerable because mature sperm have limited capacity for strand-break repair. Oxidative stress can therefore translate into base oxidation and single- or double-strand breaks, manifesting as increased SDF. Elevated SDF has been associated with poorer fertilization and embryo development and, in some studies, with adverse clinical outcomes after assisted reproduction, although effect sizes vary across assays and populations [11,17].

Several redox-linked cell-death and stress programs are being explored as additional mechanisms. Ferroptosis (iron-dependent lipid peroxidation with failure of glutathione peroxidase-4 defenses) has been reported in semen studies and has been proposed as a contributor to oxidative stress-associated male infertility. At present, this literature is best viewed as hypothesis-generating, with limited interventional data in prostatitis phenotypes [33,34].

Redox regulation in semen is not simply a matter of suppressing ROS, because physiological redox signaling is involved in capacitation. Peroxiredoxins help buffer this signaling but may be overwhelmed in inflammatory semen [11,35]. At a systems level, oxidation–reduction potential (ORP) provides an integrated measure of redox balance, and oxidative stress has also been linked to epigenetic dysregulation of spermatogenesis in emerging human and translational work [36,37].

From a management standpoint, identification of oxidative stress should trigger evaluation and treatment of upstream inflammatory and infectious drivers (culture and/or nucleic acid amplification testing when indicated) and supports time-limited, biology-aligned antioxidant and lifestyle interventions rather than prolonged empiric supplementation [38].

### 3.3. The Prostatic and Seminal Microbiome

Sequencing studies support the concept that the male reproductive tract contains a low-biomass microbial community and that its composition differs across men with prostatitis symptoms and/or abnormal semen parameters. Multiple cohorts report associations between specific taxa patterns and semen quality, particularly motility and morphology, but confounding and low-biomass sampling challenges mean that clinical causality remains uncertain. The proposed “androbactome” framework integrates gut, urinary, and seminal niches and provides a hypothesis-generating model for microbiome–endocrine–immune interactions relevant to male fertility [8,39]. Genetic-instrument (e.g., Mendelian randomization) studies suggest possible causal links for selected genera, but these inferences still require clinical validation and intervention studies before they are used to justify routine microbiome-directed care [8,39].

Microbes and their products may nevertheless contribute to sperm dysfunction through several plausible routes. In vitro studies show that common Gram-negative uropathogens can adhere to sperm, reduce progressive motility, disrupt mitochondrial function, and increase oxidative injury. More recently, toxins associated with dysbiotic female-tract communities (e.g., lipopolysaccharide and vaginolysin) have been shown to perturb capacitation-related pathways and impair fertilization in experimental models. These data support biological plausibility, but they do not by themselves establish that microbiome modulation improves fertility outcomes in prostatitis [40,41].

Chronicity and treatment resistance may be promoted by biofilm formation within prostatic ducts and by anatomic niches that limit antimicrobial penetration. Reviews of diagnostic strategies emphasize combining optimized culture with molecular testing to improve detection of fastidious or occult pathogens in symptomatic or inflammatory cases [38]. Reports that low-intensity shock-wave therapy is accompanied by shifts in prostatic or gut microbial signatures and that semen microbiome–metabolome profiles track semen quality remain exploratory and should be interpreted as hypothesis-generating adjuncts rather than outcome-grade evidence [42,43,44]. In clinical practice, semen features suggestive of outflow impairment (very low volume, acidic pH, absent fructose) or relevant imaging abnormalities should prompt evaluation for ejaculatory duct obstruction, as this represents a potentially correctable contributor to male infertility.

### 3.4. Ductal Obstruction and Stromal Fibrosis

Structural sequelae may occur in a subset of men with prolonged prostatic inflammation. Chronic cytokine exposure is associated with fibroblast activation, collagen deposition, and stromal fibrosis, which can narrow ducts and alter semen outflow. Transforming growth factor-β and TNF-α signaling are implicated in pro-fibrotic programs, and prospective clinical data suggest that metabolic traits may correlate with greater periurethral fibrosis [43,44,45]. These observations support a pathway in which persistent inflammation may contribute to a mechanical component of subfertility in selected cases.

Ejaculatory duct obstruction is an important, although relatively uncommon, clinical endpoint. Men typically present with low ejaculate volume and biochemical features of reduced seminal vesicle contribution (e.g., low fructose), and imaging may show seminal vesicle dilation, midline cysts, or ductal calcifications. In carefully selected patients, vesiculoscopy or transurethral resection of the ejaculatory ducts can improve semen parameters and symptoms, but success depends on the level and extent of scarring and recurrence can occur [46,47].

Experimental prostatitis models suggest that anti-inflammatory and anti-fibrotic interventions can reduce collagen deposition and limit ductal obliteration, and observational work raises the possibility that optimizing metabolic health may be relevant to fibrosis burden. However, translation to fertility outcomes in humans remains limited, so these concepts should primarily guide vigilance for obstruction and motivate timely evaluation when suggestive clinical features are present [43,48].

### 3.5. Psychosocial Factors and Sexual Dysfunction

CP/CPPS can affect fertility through sexual dysfunction and behavioral factors in addition to semen biology. Pain, pelvic floor dysfunction, and psychosocial distress are common and are associated with erectile dysfunction, ejaculatory disturbance, and reduced libido. Reduced intercourse frequency and avoidance of ejaculation can therefore meaningfully lower the probability of natural conception, even when semen parameters are only mildly affected [49,50,51].

Multimodal, biopsychosocial management can improve these domains. Pelvic floor re-education (including biofeedback and structured home training) has been shown to reduce symptom burden in men with chronic pelvic pain, and psychological interventions added to standard therapy can improve pain and sexual outcomes in randomized and prospective studies [52,53].

Sexual-medicine interventions can be integrated within phenotype-directed care. Phosphodiesterase type 5 (PDE5) inhibitors improve erectile function and may enhance sexual confidence and intercourse frequency in selected men, while UPOINT-guided management targets six domains (urinary, psychosocial, organ-specific, infection, neurologic/systemic, tenderness) to ensure that urinary, psychosocial, organ-specific, and tenderness components are addressed alongside infection or inflammation when present [54,55].

When prostatitis is accompanied by documented infection or clinically relevant dysbiosis, partner evaluation and coordinated couple-based management should be considered to reduce reinfection risk and potential female reproductive morbidity [56].

## 4. Female Reproductive Consequences of Male Prostatitis

Seminal plasma exposure modulates female reproductive tract immune responses and endometrial receptivity. Because prostatitis is associated with a more inflammatory seminal profile (including higher IL-6, IL-8, and TNF-α) and altered accessory-gland secretions, it may contribute, in some couples, to a more pro-inflammatory vaginal–cervical–endometrial response after intercourse and potentially to reduced receptivity. Human transcriptomic studies and mechanistic syntheses show that seminal plasma can reprogram endometrial gene expression, but direct clinical outcome data linking male prostatitis to implantation or live-birth endpoints remain limited and confounded by coexisting male and female factors [20,57,58].

Seminal EVs represent another route for male-to-female signaling, delivering proteins, lipids, and microRNAs that can modulate cervical and endometrial immune pathways and markers of receptivity in emerging human and experimental studies. In prostatitis, EV cargo may reflect an inflamed glandular origin and could plausibly shift these signals, although fertility-relevant clinical endpoints have not yet been established [59,60,61,62,63].

The semen microbiome and transmissible pathogens provide a clearer couple-level diagnostic interface. Semen dysbiosis and urogenital infections can be shared between partners, and a randomized controlled trial reported lower recurrence of bacterial vaginosis when male partners were treated alongside standard female therapy. Established sexually transmitted pathogens (e.g., *Chlamydia trachomatis* and *Mycoplasma genitalium*) can be asymptomatic reservoirs in men yet cause cervicitis, pelvic inflammatory disease, and tubal-factor infertility in women, supporting coordinated partner evaluation and treatment when indicated [56,64,65,66].

Female-partner evaluation should therefore be selective, risk-based, and time-sensitive. When there is evidence of male tract infection or inflammatory semen features and/or recurrent vaginal symptoms, guideline-aligned nucleic acid amplification testing for chlamydia and gonorrhea is appropriate according to risk, with trichomoniasis and Mycoplasma genitalium testing considered in persistent cervicitis or pelvic inflammatory disease. In women with recurrent bacterial vaginosis who are attempting conception or ART, clinicians can discuss the evidence for partner therapy and consider combined male oral and topical regimens alongside standard female treatment. In couples with recurrent implantation failure or recurrent pregnancy loss without structural or genetic explanations, evaluation for chronic endometritis is reasonable (hysteroscopy plus endometrial plasma-cell immunohistochemistry). Endometrial or vaginal microbiome testing remains investigational; comparative analyses show imperfect concordance with hysteroscopy and CD138 staining and do not define actionable thresholds that improve live birth. Where used, such testing should be limited to research protocols or highly selected cases and should not delay standard infertility evaluation or indicated assisted reproductive technology (ART) timelines [56,65,67].

Overall, male prostatitis may have couple-level reproductive implications through inflammatory, vesicular, and microbial exposure, but most links remain associative and should be framed as biologically plausible rather than definitive. Diagnostic and management priorities are therefore to identify and treat proven infections, coordinate partner care when needed, and proceed with standard infertility evaluation and appropriate ART timing rather than pursuing investigational testing in routine practice.

## 5. Diagnostic Approach to Prostatitis-Related Male Infertility

A clinically useful evaluation anchors each test to a mechanistic axis that is plausibly perturbed by prostatic inflammation, namely inflammatory activity, oxidative stress, microbial burden, and outflow obstruction. Framing the workup in this way reduces redundant testing, links results to treatment choices, and aligns with contemporary translational insights that connect cytokine excess, redox imbalance, dysbiosis, and ductal remodeling to semen dysfunction [68]. Table 2 summarizes a pragmatic, phenotype-driven investigation panel that links each recommended test to these mechanistic axes and highlights how results can be translated into targeted management decisions.

### 5.1. Semen Analysis—Leukocytes, Viscosity, and Liquefaction

The semen examination remains the gateway test yet should be interpreted through an inflammatory lens. Quantification of round cells and peroxidase-positive leukocytes is essential because leukocytospermia is defined as ≥1.0 × 10^6^ white blood cells (WBC)/mL and signal an activated innate response that predicts both ROS generation and cytokine enrichment. Contemporary syntheses reaffirm leukocytospermia as a reliable proxy for male accessory gland inflammation and a frequent accompaniment of bacteriospermia in symptomatic and subfertile men [6]. A focused review from the past year underscores the clinical salience of this finding by detailing links to impaired motility, viability, and sperm DNA injury and by outlining management contingencies once inflammatory etiology is documented [9]. Semen rheology deserves equal attention. Hyperviscosity and delayed liquefaction arise when prostatic proteolytic activity is reduced and often coexist with elevated seminal pH and altered biochemical markers such as zinc or acid phosphatase, patterns that point toward a prostatic source of inflammation and that correlate with oxidative stress in translational studies [6]. Routine semen parameters can be normal in many men with CP/CPPS, yet a sizeable minority display reduced progressive motility and abnormal morphology, findings that should prompt deeper interrogation of inflammatory and oxidative axes rather than premature reassurance [6].

### 5.2. Seminal Cytokine Biomarkers

Inflammatory biomarkers in seminal plasma add mechanistic resolution when leukocytes or clinical features raise suspicion. Among measured mediators, IL-8 is the best supported surrogate of genital tract inflammation, with robust inverse associations with motility and consistent elevation in infertile cohorts. Two recent Cytokine reports using standardized multiplex or flow cytometric approaches confirm higher seminal IL-6 and IL-8 in men with semen abnormalities and document negative correlations between IL-8 and motility across analytic platforms [18,19]. A scoping analysis of immune-regulatory cytokines in healthy men highlights the wide physiological range of seminal cytokine values, a crucial caveat that argues for interpreting IL-8 within a panel or alongside inflammatory clinical context rather than as a solitary diagnostic [20]. When reported, cytokine results should specify assay platform and units (typically pg/mL) to support longitudinal within-man monitoring and cross-study comparability. In practice, an elevated IL-8 result in an inflammatory semen phenotype strengthens the case for targeted microbiology and for parallel assessment of oxidative stress.

### 5.3. Oxidative Stress Assessment

Because excessive oxidant generation is a principal effector of sperm damage in inflammatory states, direct measurement of seminal oxidative stress is informative and actionable. Flow-cytometric and luminometric assays for ROS quantify oxidant output in real time and reveal excess production in a meaningful fraction of subfertile men, including those with bacteriospermia, patterns that are not captured by routine semen variables alone [69]. ORP can provide an integrated index of redox balance, typically reported as mV/10^6^ sperm/mL when normalized to sperm concentration. A recent meta-analysis demonstrates higher ORP in infertile men with abnormal semen quality and supports its diagnostic discrimination, while a contemporary andrology study cautions that static ORP may be unhelpful in idiopathic infertility when measured in isolation [70,71]. In MiOXSYS-based studies, example normalized ORP cutoffs around 1.34–1.36 mV/10^6^ sperm/mL have been proposed to discriminate normal from abnormal semen quality; however, thresholds are device-, processing-, and lab-specific and should be locally validated [72,73]. These complementary findings favor a pragmatic approach in which laboratories select a validated ROS platform, report total antioxidant capacity (TAC) or ORP as a context measure, and interpret results against clinical and inflammatory cues rather than fixed universal thresholds. Mechanistic reviews from 2025 consolidate the rationale by detailing how leukocyte-derived oxidants drive lipid peroxidation, protein modification, mitochondrial dysfunction, premature capacitation, and nuclear injury in sperm and by identifying peroxiredoxins as gatekeepers that buffer physiological redox signaling during capacitation [74,75].

### 5.4. Sperm DNA Fragmentation Testing

Assessment of sperm chromatin integrity closes the loop between inflammation-driven oxidative stress and reproductive outcomes. The American Urological Association and the American Society for Reproductive Medicine advise against routine DNA fragmentation testing at the first visit yet support its use in defined scenarios such as recurrent pregnancy loss (e.g., SCSA for DFI-based risk stratification), repeated IVF/ICSI failure (e.g., TUNEL for direct strand breaks or Comet for single-cell profiling), unexplained infertility (SCSA or TUNEL depending on the clinical question), and when oxidative or inflammatory factors are suspected (e.g., TUNEL or Comet in men with high oxidative stress or persistent leukocytospermia) [10]. Method choice should follow laboratory capability and clinical question. The terminal deoxynucleotidyl transferase dUTP nick end labeling (TUNEL) assay detects strand breaks directly, the sperm chromatin structure assay quantifies susceptibility to denaturation, and the comet assay offers single-cell resolution; all correlate variably with fertilization competence. Recent reviews and outcome studies link higher DNA fragmentation to miscarriage and adverse perinatal metrics in assisted reproduction, providing clinically relevant end points for men with inflammatory semen profiles [17,76,77]. When DNA fragmentation is increased in the setting of documented oxidative stress or inflammation, results can guide antioxidant or anti-inflammatory interventions, reinforce antimicrobial stewardship when infection is proven, and inform selection of assisted reproductive strategies.

### 5.5. Microbiological Testing

Microbiological testing is the hinge between inflammatory biomarkers and targeted therapy. When prostatitis is suspected, microbiology should aim not only to detect organisms but also to localize the site of infection. The Meares–Stamey four-glass test (VB1, VB2, expressed prostatic secretion, and post-massage urine VB3) is the reference localization method underpinning NIH categorization, with the simplified two-glass pre-/post-massage test serving as a pragmatic alternative in routine practice [78,79]. In infertility evaluation, semen culture is valuable because it samples the ejaculate directly and provides antimicrobial susceptibility; however, semen culture alone does not substitute for localization testing, as it cannot distinguish urethral contamination from true prostatic infection. Quantitative thresholds around 10^3^ colony-forming units (CFU)/mL are commonly used to define clinically meaningful bacteriospermia in the prostatitis/semen-culture literature, while acknowledging contamination risk in low-biomass specimens [79,80]. A fertility-oriented adaptation is the “five-sample” concept (four-glass localization plus an ejaculate culture as an added specimen), which has been proposed to increase diagnostic yield for traditional uropathogens compared with localization testing alone in chronic bacterial prostatitis cohorts [79].

At the same time, culture sensitivity is limited for fastidious or intracellular organisms and for infections embedded in prostatic biofilms. Nucleic acid amplification tests therefore expand detection to sexually transmitted pathogens such as *Chlamydia trachomatis* and *Mycoplasma genitalium* and improve yield when leukocytospermia or elevated IL-8 coexists with negative cultures [38,81]. Combining optimized culture (including localization when feasible) with NAAT maximizes accuracy, permits antibiotic selection when a pathogen is recovered, and reduces indiscriminate antimicrobial exposure when none is found [6,38]. For refractory, culture-negative cases with persistent inflammatory signatures, advanced microbial profiling can be selectively deployed. High-depth amplicon sequencing and metagenomic approaches reveal low-abundance communities and have demonstrated associations between dysbiotic semen microbiota and abnormal motility or morphology in men under evaluation for infertility, while integrated microbiome-metabolome profiling has begun to map microbe-metabolite patterns that track semen quality. These methods are hypothesis-generating and should not be routine, yet they are increasingly valuable in difficult diagnostic scenarios and in research settings that aim to personalize therapy [38,82,83].

### 5.6. Imaging and Assessment of Obstructive Factors

An anatomic pass is required whenever low ejaculate volume, acidic semen, absent fructose, or a history suggestive of ductal disease raises concern for obstruction. Transrectal ultrasonography can demonstrate midline cysts, calcifications, or seminal vesicle dilatation, and magnetic resonance imaging can refine equivocal findings. Evidence summaries place ejaculatory duct obstruction as an infrequent but treatable cause of male infertility, with transurethral resection or vesiculoscopy improving outflow in selected men [84]. Recent case-based work illustrates partial and functional obstruction patterns in young men, underscoring the value of imaging in persistent azoospermia or severe oligozoospermia with preserved testicular function [85]. Emerging prospective data further connect systemic metabolic traits to peri-urethral prostatic fibrosis, hinting at modifiers of stromal remodeling that may intersect with chronic inflammation in the prostate [43].

### 5.7. Proposed Stepwise Diagnostic Algorithm

**Figure 1:**
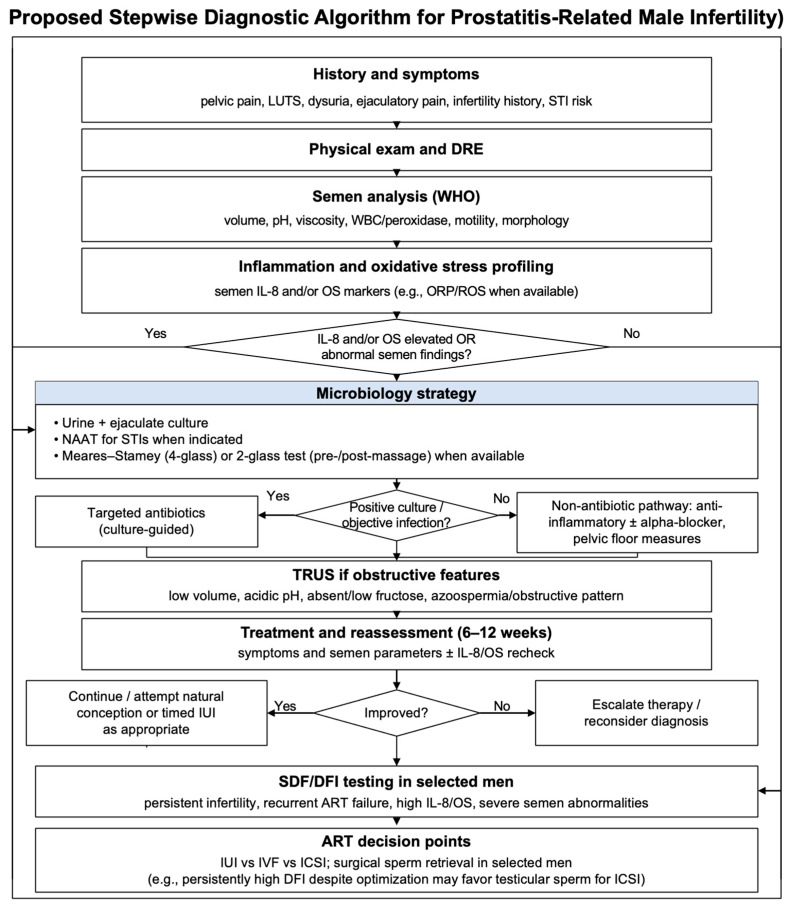
Proposed stepwise diagnostic algorithm for suspected prostatitis-related male infertility, emphasizing infection localization testing (Meares–Stamey four-glass/two-glass; infertility: add ejaculate culture as an additional specimen) and reassessment at 6–12 weeks after targeted therapy. Where thresholds are shown, leukocytospermia is defined as ≥1.0 × 10^6^ WBC/mL; IL-8 is reported in pg/mL (assay-dependent); ROS units are platform-specific (report with stated normalization); and ORP is reported as mV/10^6^ sperm/mL (example MiOXSYS discriminator ~1.34–1.36 mV/10^6^ sperm/mL; device-/lab-specific). In selected cases with persistently high DFI despite optimization, consideration may be given to testicular sperm for ICSI. Abbreviations: ART, assisted reproductive technology; DFI, DNA fragmentation index; DRE, digital rectal examination; ICSI, intracytoplasmic sperm injection; IL-8, interleukin-8; IUI, intrauterine insemination; IVF, in vitro fertilization; LUTS, lower urinary tract symptoms; NAAT, nucleic acid amplification test; ORP, oxidation–reduction potential; OS, oxidative stress; pH, potential of hydrogen; ROS, reactive oxygen species; SDF, sperm DNA fragmentation; STI, sexually transmitted infection; TRUS, transrectal ultrasound; WBC, white blood cells; WHO, World Health Organization.

In practice, a coherent, stepwise pathway avoids scattershot testing. The evaluation begins with a detailed history, symptom scoring, and focused examination to define pain, urinary, sexual, and systemic features that raise suspicion for prostatic inflammation. Semen analysis is then interpreted with attention to leukocytes (≥1.0 × 10^6^ WBC/mL), viscosity, liquefaction, pH, and accessory gland markers to flag an inflammatory pattern. The inflammatory and oxidative axes are profiled through seminal IL-8 and a validated oxidative stress platform (ROS and/or ORP), with sperm DNA fragmentation added when clinical criteria or redox results indicate increased reproductive risk. When prostatitis is suspected, microbiology should make localization testing central (preferably the Meares–Stamey four-glass test; a two-glass pre-/post-massage test when the four-glass is impractical), paired with quantitative cultures and susceptibility; in infertility evaluation, adding an ejaculate culture as a fifth specimen (“five-sample” concept) can increase diagnostic relevance. NAAT for sexually transmitted organisms is added when inflammation persists with negative cultures or when risk factors or partner concerns exist. Imaging with transrectal ultrasonography is reserved for suggestive biochemistry, very low volume, or semen features that imply obstruction, and advanced sequencing is held for refractory, culture-negative inflammation when results would plausibly redirect therapy. Findings are integrated to guide antibiotics when pathogens are proven, anti-inflammatory and antioxidant strategies are used when redox and cytokine signatures dominate, and assisted reproduction choices are used when DNA fragmentation remains high despite optimization. Repeat semen analysis (and relevant biomarkers/cultures) at 6–12 weeks after targeted therapy closes the loop and supports escalation to reproductive technologies when indicated; for example, persistently high DFI/SDF despite optimization may prompt consideration of testicular sperm for ICSI in selected cases [6,10,19,69,70,82]. Figure 1 summarizes this diagnostic and treatment-escalation pathway and highlights the key decision points that link test results to targeted management.

## 6. Therapeutic Strategies and ART Interface

### 6.1. Infection and Inflammation

Effective management of prostatitis-related infertility begins with eradicating identifiable infections and quelling inflammation in the prostate [86]. In acute category I and chronic bacterial prostatitis category II, culture-guided antibiotic therapy is the cornerstone. Identification of uropathogens enables targeted treatment, commonly a four to six week course of a fluoroquinolone or trimethoprim sulfamethoxazole, to eradicate infection and reduce seminal leukocytes [87,88,89,90]. Empiric antibiotic trials may also be considered in select chronic pelvic pain syndrome cases (category III) when inflammation is present (elevated leukocytes or interleukins) [91,92]. When antibiotics are trialed in CP/CPPS, therapy should be guided by cultures and/or NAATs when possible and limited to the shortest effective course to support antimicrobial stewardship [38]. Adjunctive anti-inflammatory therapy is recommended to disrupt the vicious cycle of cytokine-driven injury [93,94,95]. Nonsteroidal anti-inflammatory drugs and phytotherapeutics (e.g., quercetin or pollen extracts) have shown symptomatic benefit in chronic prostatitis, likely by dampening prostaglandin and interleukin activity [96,97,98]. By reducing prostatic pain and swelling, these agents can secondarily improve ejaculatory function and semen parameters (motility, viscosity) that are impaired by inflammation [99,100]. Ultimately, a multimodal approach is favored in chronic prostatitis to address all relevant domains of the condition.

The UPOINT system supports individualized therapy. For example, treatment may combine alpha blockers for voiding symptoms, pelvic floor physical therapy for muscular tension, and neuromodulators for pain, alongside antimicrobials and anti-inflammatory agents [101,102,103]. Such phenotype-tailored regimens yield high response rates in CP/CPPS and have been credited with restoring sexual function and intercourse frequency in affected men [99,103,104]. This comprehensive control of infection, pain, and inflammation removes key impediments to natural fertility, since a quiescent prostate can then support normal semen liquefaction, reduce spermatotoxic leukocyte products, and allow more regular, pain-free ejaculation [105].

### 6.2. Oxidative Stress

Inflammatory prostatitis exposes sperm to sustained leukocyte-derived ROS, lipid peroxidation, and oxidative DNA damage. Therapeutic strategies therefore aim not only to treat the underlying inflammation but also to rebalance redox homeostasis and safeguard sperm function. Antioxidant supplementation has become a mainstay adjunct for men with prostatitis-related oxidative stress. A broad range of agents, including vitamin C, vitamin E, carnitine such as L-carnitine and L-acetylcarnitine, coenzyme Q10, N acetylcysteine, lycopene, selenium, and omega 3 fatty acids, have shown potential to improve sperm concentration, motility, and morphology and, in many studies, to reduce SDF [36,106]. These compounds augment seminal antioxidant capacity and help counter the peroxidative and nuclear injury characterizing inflammatory semen [36,106].

Antioxidant therapy is best viewed as a time-limited, biology-aligned intervention rather than a quick fix. Because a full spermatogenic cycle spans roughly 74 days (~10–11 weeks), supplements are usually prescribed for an initial 6–12-week period (≈one cycle), with reassessment thereafter [107]. Experts emphasize tailoring regimens to the individual, using baseline oxidative stress metrics where available, and combining supplementation with lifestyle modification (diet, weight, smoking, environmental toxins) to reduce upstream oxidant load [36,106]. A 2024 network meta-analysis and updated infertility guidelines support the use of selected antioxidants in men with objectively high oxidative stress, noting improvements in motility and DFI after ~3–6 months (i.e., one to two cycles) of therapy [10,108].

Antioxidants should be used as an adjunct to, rather than a replacement for, targeted management of infection and inflammation. In practice, men with prostatitis and elevated oxidative stress markers may be offered a rational regimen such as carnitine with vitamin E alongside treatment of prostatitis drivers. Follow up semen analysis with repeat ROS or ORP testing and SDF assessment when indicated can prevent prolonged empiric supplementation. If oxidative indices normalize with improvement in semen quality, couples may continue attempts at natural conception or consider less invasive assisted reproduction. If redox markers remain abnormal despite optimized care, escalation to more advanced interventions may be appropriate [17,69,70].

### 6.3. Microbiome

Recognition of a prostate and seminal microbiome has expanded the therapeutic framework for prostatitis-related infertility. When bacteriospermia is identified, management should be pathogen-directed and judicious. Standard semen culture, using a threshold of approximately 10^3^ CFU/mL to define significant growth, remains essential for guiding antibiotic choice and is complemented by nucleic acid amplification testing for fastidious sexually transmitted organisms, including *Chlamydia trachomatis* and *Mycoplasma genitalium* [109,110]. This combined microbiologic approach improves pathogen detection and supports tailored antibiotic selection informed by susceptibility data, while limiting broad spectrum use when results are negative [38]. Antimicrobial stewardship is critical because unnecessary antibiotic exposure in chronic prostatitis can promote resistance and disrupt the genital microbiota without clear benefit. Antibiotics should therefore be reserved for confirmed infection, matched to the organism identified, and limited to the shortest effective duration. Examples include fluoroquinolones for an identified Gram-negative rod and doxycycline or azithromycin for an identified atypical organism. When *Chlamydia trachomatis* or *Mycoplasma genitalium* is detected, coordinated treatment of the sexual partner is essential to reduce reinfection risk and protect the female reproductive tract [65,66,111].

Beyond classic pathogens, increasing attention is being directed toward correcting dysbiosis, defined as shifts in microbial community structure that are associated with CP [112]. Early clinical research has turned to probiotics and prebiotics as therapeutic tools to modulate the male reproductive microbiome. A recent systematic review of randomized trials (2024) found that probiotic supplementation in idiopathic infertile men yielded significant improvements in all semen parameters, particularly motility, while also lowering oxidative stress and sperm DNA damage [113]. The probiotics, commonly *Lactobacillus* and *Bifidobacterium* strains and sometimes combined with prebiotic fiber as synbiotics, likely exert benefit by outcompeting pro-inflammatory bacteria and producing metabolites that support sperm health [114,115]. For example, multi-strain probiotic regimens have been shown to increase TAC of semen and decrease pro-inflammatory cytokines like TNF-α and C-reactive protein (CRP) in men with poor sperm quality [114,116,117]. Although still an emerging area and not yet guideline-supported for prostatitis-related infertility, these findings suggest a potential adjunctive role for probiotics or dietary prebiotics in selected patients. Importantly, these adjuncts are used alongside, not instead of, pathogen-directed care. Men with persistent inflammatory prostatitis symptoms despite negative routine cultures may also be considered for advanced microbiome profiling, including 16S rRNA gene sequencing or metagenomics, in research settings to identify occult bacterial communities or biofilm-associated drivers of inflammation [112,118]. Although these high depth analyses are not yet part of routine care, they have linked seminal dysbiosis with impaired sperm motility and may support more individualized management in the future [82]. In parallel, nonantibiotic strategies aimed at disrupting persistent microbial communities are under investigation. Low-intensity shockwave therapy, used for pain in CP/CPPS, has been reported to shift prostatic and gut microbiota profiles, raising the possibility that biomechanical interventions could mitigate microbiome-associated inflammation [42,119].

### 6.4. Integration with Assisted Reproductive Technologies

A crucial component of managing prostatitis-associated male infertility is deciding when and how to incorporate ART. Clinical counseling is central throughout this process. Men should be advised that seminal inflammation, as indicated by leukocytospermia, and elevated SDF are associated with poorer reproductive outcomes, including reduced natural fertility and lower success rates with assisted reproduction. For instance, elevated seminal white blood cells often coincide with oxidative sperm damage; accordingly, infertile men with leukocytospermia are counseled about the potential negative impact of inflammation on sperm DNA integrity and embryo development. Similarly, an abnormally high DFI predicts higher miscarriage rates and even adverse perinatal outcomes after IVF/ICSI, underscoring the importance of optimizing sperm DNA quality before attempting conception [76,120]. These discussions set realistic expectations and reinforce why aggressive management of the prostatitis is pursued prior to ART.

After completion of male-focused therapy, typically over several months with antibiotics, anti-inflammatory agents, and antioxidants as indicated, the couple’s fertility plan should be reassessed [10]. If semen parameters improve substantially, such as resolution of leukocytospermia with normalization of motility and DNA fragmentation, attempts at natural conception or less invasive options such as intrauterine insemination may be reconsidered [10]. However, persistent abnormalities despite optimization prompt a timely transition to assisted reproduction [10]. In clinical practice, couples rarely postpone childbearing until the semen profile is fully normalized, particularly when female age or ovarian reserve makes time a limiting factor [10].

Management is therefore pragmatic. After reasonable opportunities for male optimization have been completed, proceeding to IVF is often appropriate even if low grade inflammatory or oxidative markers persist. Laboratory techniques can mitigate residual effects. Sperm washing and density gradient preparation remove most leukocytes and ROS and intracytoplasmic sperm injection can bypass impaired motility or antisperm antibodies by injecting a selected sperm directly into the oocyte [121,122,123,124].

ICSI has largely replaced conventional IVF in severe male-factor infertility and is commonly used in men with severe teratozoospermia or asthenozoospermia because it consistently achieves fertilization despite functional sperm compromise [10]. The sperm selected for ICSI are typically the most motile and morphologically normal cells available and are therefore more likely to carry lower levels of DNA damage, which can partly mitigate residual oxidative stress within the ejaculate. However, when the male partner has persistently and markedly elevated DNA fragmentation, additional measures may be considered while assisted reproduction proceeds. Options include the use of testicular sperm for ICSI, which can show lower DNA fragmentation than ejaculated sperm in settings of chronic inflammation, and the application of advanced sperm selection techniques such as magnetic activated cell sorting to reduce apoptotic sperm enrichment [125,126,127,128]. These adjunctive strategies illustrate a tailored approach to optimize outcomes when oxidative stress remains a relevant contributor.

Throughout ART planning, the reproductive urologist and fertility specialist work in tandem [68]. Care for the male partner is aligned with the female partner’s ovarian stimulation schedule so that sperm quality is optimized by the time of oocyte retrieval. If prostatitis recurs or semen parameters deteriorate, banking higher quality samples can protect against subsequent variability [129]. Integration of ART therefore depends on careful sequencing that links diagnosis to intervention. After targeted treatment and follow up assessment, escalation to IVF or ICSI is undertaken when indicated [10]. This approach of treatment followed by reassessment and then assisted reproduction is supported by recent prospective studies reporting higher pregnancy rates when male inflammatory contributors are addressed before ART rather than proceeding without optimization [130,131].

## 7. Knowledge Gaps, Research Priorities, and Translational Opportunities

Although mechanistic and clinical associations between prostatitis and impaired fertility are increasingly recognized, the available clinical literature remains dominated by small, heterogeneous, and often cross-sectional or retrospective studies. To move beyond association and toward actionable care pathways, the field needs prospective longitudinal cohorts and adequately powered multicenter randomized trials that prespecify standardized, couple-relevant fertility outcomes and harmonized laboratory definitions.

### 7.1. Standardizing Clinical Endpoints in Prostatitis-Related Infertility Trials

A critical gap in current research is the lack of standardized, fertility-centered endpoints in studies evaluating interventions for prostatitis-associated male infertility. Many reports prioritize intermediate outcomes like semen parameters, whereas only about half of recent infertility trials report pregnancy rates and even fewer report live birth outcomes [132]. This heterogeneity limits comparability weakens meta-analysis and makes it difficult to translate biomarker or semen improvements into couple-level benefits. Future studies should be prospectively designed and, where feasible, conducted as adequately powered multicenter randomized trials, with time-to-pregnancy and live birth as preferred primary endpoints (and standardized reporting of clinical pregnancy and miscarriage as core secondary outcomes). To ensure interpretability across centers, semen and biomarker outcomes should also be standardized as secondary endpoints using harmonized methods (e.g., WHO semen analysis with leukocyte quantification and agreed assay platforms/thresholds for oxidative stress and sperm DNA fragmentation). Establishing and adopting a core outcome set for male infertility trials is therefore a priority [132].

### 7.2. Validation of Inflammatory and Oxidative Biomarkers as Theranostic Tools

Existing evidence indicates that seminal IL-6 and IL-8, oxidative stress measures including ROS, TAC, and ORP, and the sperm DFI are consistently altered in men with prostatitis and correlate with semen quality [133]. Elevated IL-8 and leukocytospermia align with accessory gland inflammation, increased oxidative stress markers accompany reduced motility and membrane injury, and higher DNA fragmentation is associated with poorer fertilization, embryo competence, and live birth outcomes in assisted reproduction [134,135,136]. Despite biological plausibility and increasing clinical uptake, these biomarkers remain adjunctive and are applied inconsistently across settings.

A major research priority is to advance from association to validated theranostic use. Prospective and adequately powered studies are needed to relate baseline IL-8, oxidative stress measures, and DNA fragmentation to reproductive endpoints such as time to pregnancy, live birth, and miscarriage in couples where the male partner has prostatitis. Studies should also determine whether treatment-related changes in these markers predict benefit. For instance, it remains unclear whether reductions in IL-8 or ROS after antimicrobial and anti-inflammatory therapy translate into higher natural conception rates or improved IVF outcomes, as well as whether declines in DNA fragmentation provide a reliable surrogate for clinical improvement [11].

Methodological standards also require clarification. Thresholds for abnormal IL-8, oxidative stress, and DNA fragmentation vary across assays and laboratories. Establishing clinically meaningful cutoffs or percentile-based risk strata is necessary before these tests can guide decisions on treatment duration, timing of assisted reproduction, or selection of ejaculated versus testicular sperm. Cost effectiveness and access, particularly in low-resource settings, also warrant attention so that biomarker-guided care does not widen disparities. Future trials should embed these measures within standardized diagnostic pathways, report core fertility outcomes, and prespecify how biomarker data will be used for stratification or treatment tailoring. With these steps, IL-8, oxidative stress measures, and DNA fragmentation can progress from promising research tools to practical guides for individualized management of prostatitis-related infertility [18].

### 7.3. Microbiome-Targeted Interventions and Semen Quality

Increasing evidence links the male genitourinary microbiome to prostatitis and sperm health, but controlled trials are needed to clarify if modulating this microbiome can improve fertility. Antibiotic therapy is standard for chronic bacterial prostatitis and has been reported to not only eradicate infection but also significantly improve sperm parameters in some cases [137]. For instance, appropriate antibiotic treatment of confirmed bacterial prostatitis can restore ejaculate volume and motility in affected men [137]. Beyond classical antibiotics, probiotic and synbiotic interventions represent a forward-looking approach. These aim to restore a healthy urogenital and gut microbiome, thereby reducing prostatic inflammation. Early trials are encouraging. Adjunct probiotic therapy, such as *Enterococcus faecium* and *Saccharomyces boulardii* administered during a fluoroquinolone course, has been associated with higher infection clearance in male accessory gland infection, with eradication reported in 76% of men receiving probiotics plus antibiotics compared with 50% receiving antibiotics alone [138]. Probiotics have also been associated with faster symptom resolution and longer remission in chronic prostatitis patients [139]. What remains unknown is whether these microbiome-targeted therapies tangibly improve semen quality or fertility outcomes.

Future research should prioritize randomized controlled trials comparing antibiotics with a placebo in asymptomatic inflammatory prostatitis and probiotics or synbiotics with a placebo in both bacterial and nonbacterial prostatitis, with outcomes that include sperm concentration, motility, DNA integrity, and pregnancy rates. Such studies would help define the causal contribution of microbial dysbiosis to male infertility and could support the development of adjunctive treatments, including strain-selected probiotic formulations that complement standard therapy. Work is also needed to determine optimal timing, including administration alongside antibiotics or as a standalone intervention, and to evaluate combination approaches that incorporate prebiotics or postbiotics to optimize the genitourinary microbiome. Given the proposed links between the gut and prostate in chronic pelvic inflammation, microbiome-targeted strategies represent a plausible translational avenue to improve the prostatic milieu and reproductive potential.

### 7.4. Extracellular Vesicles and Prostasomes as Inflammation Biomarkers

The prostate secretes abundant EVs, commonly known as prostasomes, into semen. These vesicles carry proteins, lipids, and RNA that influence sperm function, and they present a frontier for both diagnostics and understanding pathophysiology. In normal semen, prostasomes support fertility. Seminal exosomes from healthy men have been shown to enhance sperm motility and capacitation, and specific EV fractions can reduce sperm ROS by delivering antioxidant enzymes [140]. In the context of prostatitis, however, EV cargo and effects may be markedly altered. It is hypothesized that inflamed prostatic tissue releases EVs enriched in inflammatory mediators (such as IL-1β, IL-8, TNF-α) and danger signals. These vesicles can traffic to neighboring cells (including sperm and the female reproductive tract), potentially propagating inflammation or impairing sperm function. Recent findings in a prostatitis model indicate that EVs can indeed carry cytokines and chemokines to target cells, activating inflammatory signaling pathways and promoting immune cell infiltration in prostate tissue [141]. Such EV-mediated communication might sustain local inflammation and contribute to tissue damage and could also negatively impact sperm through surface interactions or uptake of EV content. A key research opportunity is to characterize the molecular signatures of prostatic EVs in men with chronic prostatitis (bacterial and non-bacterial) and to determine how these signatures correlate with glandular inflammation and fertility outcomes. For example, profiling EV-associated microRNAs or surface proteins in semen could yield non-invasive biomarkers reflective of prostatic immune activity. There is already precedent in other fields (e.g., prostate cancer diagnosis from urine exosome RNAs) that similar approaches could work for inflammatory prostatitis. Implementing high-throughput “omics” techniques on seminal EVs is a priority [141].

Comparative profiling of seminal EV in infertile men with prostatitis and fertile controls may reveal diagnostic signatures, including coordinated sets of inflammation-associated microRNAs that indicate subclinical prostatic inflammation with reproductive relevance. EV also provide a functional readout of the prostatic milieu. Shifts in prostasome cargo, including depletion of enzymes that support sperm function or enrichment of factors that promote oxidative stress, may directly compromise fertilizing capacity. Defining these alterations could inform therapeutic approaches that aim to restore physiologic vesicle production or attenuate harmful vesicle sperm interactions. Overall, investigation of seminal EV and prostasome profiles offers two complementary advances, biomarker development for detection and longitudinal monitoring of prostatic inflammation in infertile men, and mechanistic insight into how chronic prostatitis reshapes the sperm microenvironment.

### 7.5. Inflammasome Activation and Ferroptosis as Therapeutic Targets

Preclinical and mechanistic work implicates both NLRP3 inflammasome activation and ferroptosis in the pathogenesis of chronic prostatitis and its reproductive sequelae [142]. In rodent models, pharmacologic inhibition of NLRP3 or its upstream signals reduces prostatic cytokine production, immune-cell infiltration, pain-related behavior, and stromal fibrosis, while ferroptosis inhibitors that restore glutathione peroxidase-4 activity and limit lipid peroxidation attenuate inflammation, fibrotic remodeling, and mast-cell activation [142,143]. These studies identify discrete molecular nodes that could, in principle, be targeted to protect the prostatic microenvironment and, downstream, sperm quality.

Translating these insights into human therapeutics raises several unanswered questions. First, the extent to which inflammasome and ferroptosis pathways are activated across the clinical spectrum of prostatitis (NIH categories II–IV) remains poorly defined. Systematic profiling of semen-expressed prostatic secretions and, where feasible, tissue for IL-1β, IL-18, caspase-1 activity, NLRP3 components, glutathione peroxidase-4, iron-handling proteins, and lipid-peroxidation products is needed to map these pathways onto symptom phenotypes, semen parameters, and fertility outcomes. Such work would clarify whether a distinct subgroup of “inflammasome-high” or “ferroptosis-high” patients exists who might benefit most from targeted modulation [25].

Second, safety considerations are paramount. NLRP3 and ferroptosis play roles in host defense, tumor surveillance, and tissue homeostasis; long-term systemic inhibition could therefore carry off-target risks, particularly in reproductive-age men. Future drug development and early-phase trials will need to explore strategies that localize delivery to the prostate, use short treatment windows, or exploit context-selective modulators (for example, agents that bias inflammasome activity rather than abolish it). Fertility-focused studies should incorporate comprehensive endpoints including semen quality, DNA fragmentation, and pregnancy or live-birth rates, alongside standard pain and symptom scores [28].

There is also an opportunity to integrate inflammasome and ferroptosis pathway modulation with established treatment strategies. Clinical trials could evaluate whether adding a well-tolerated NLRP3 or ferroptosis pathway modulator to standard multimodal care, including antimicrobials, anti-inflammatory agents, antioxidants, and pelvic floor or psychological therapy, improves outcomes in clearly defined patient phenotypes. Careful patient selection, biomarker-guided enrichment, and rigorous translational correlative analyses will be required to determine whether these targets can be advanced into clinical management of prostatitis-associated infertility [33,34].

### 7.6. Design Considerations for Future Cohort Studies and Trials

To effectively address the above knowledge gaps, future research must also improve the study design and stratification of prostatitis-related infertility investigations. One recommendation is to explicitly stratify patients by the established NIH prostatitis categories (I–IV) in both observational cohorts and randomized trials [3]. Prostatitis is a heterogeneous syndrome. Acute bacterial prostatitis Type I, chronic bacterial prostatitis Type II, chronic prostatitis and chronic pelvic pain syndrome Type III with inflammatory Type IIIA and noninflammatory Type IIIB subtypes, and asymptomatic inflammatory prostatitis Type IV each have distinct etiologies and may differ in their effects on male fertility [3]. Stratification ensures that outcomes can be analyzed within more uniform subgroups; for example, an antibiotic trial in chronic bacterial prostatitis (II) can be interpreted separately from results in CP/CPPS (III), and patients with purely noninflammatory pelvic pain (IIIB) can be distinguished from those with leukocytosis (IIIA). In past studies, the failure to account for these distinctions may have obscured important differences in response to therapy or prognosis. Additionally, researchers should broaden inclusion criteria to incorporate men with “axis-positive” asymptomatic inflammation, notably NIH Category IV prostatitis. These men have no pain symptoms yet demonstrate objective evidence of prostatic inflammation, such as increased seminal leukocytes or elevated IL-8, findings that are often identified during infertility evaluation [3]. Historically, because such men do not present with complaints, they have been underrepresented in clinical studies. However, asymptomatic inflammatory prostatitis could be an occult contributor to male infertility in a subset of patients. The World Health Organization has long recognized that leukocytospermia (>1 × 10^6^ WBC/mL) can define subclinical prostatitis, and population studies find a non-trivial prevalence of asymptomatic inflammation even in healthy men [144]. Including Category IV patients in infertility research will allow determination of whether treating their inflammation (e.g., with anti-inflammatory or antimicrobial therapy) improves fertility outcomes. This is especially relevant for men with idiopathic infertility where a silent prostatic inflammation might be the missing piece. Furthermore, future trials should incorporate NIH Chronic Prostatitis Symptom Index (CPSI) scores and related phenotypic classifications (e.g., UPOINT system) to phenotype patients more granularly.

Phenotype-driven approaches can identify patient clusters, such as men with an infectious pattern or a prominent psychosocial burden, that may differ in prognosis and treatment response. In trial design, rigorous baseline characterization is essential. Key variables include duration of infertility, coexisting male factors such as varicocele or metabolic comorbidity, and female partner factors, which should be measured and incorporated through stratification or prespecified adjustment. When feasible, interventions should be evaluated in randomized placebo-controlled studies for pharmacologic agents and supplements, with adequate statistical power. Follow up should be sufficient to capture fertility endpoints, which often requires at least six to twelve months depending on the outcome assessed. A multidisciplinary design that includes urologists, andrologists, reproductive endocrinologists, microbiologists, and related specialists can ensure that studies address the full clinical and biological spectrum of prostatitis-associated infertility. Applying these principles should improve attribution of benefit to specific interventions and support translation into clinically meaningful gains in pregnancy outcomes [133].

## 8. Conclusions

Prostatitis, whether infectious or inflammatory, should be considered a potentially treatable contributor to male subfertility. In infertile men presenting with pelvic or urinary symptoms, a history of genital tract infection, or evidence of seminal inflammation such as leukocytospermia or IL-8, the diagnostic workup should extend beyond routine semen analysis to include targeted microbiological testing using semen culture with or without nucleic acid amplification methods, assessment of oxidative stress, and selective evaluation of SDF. Imaging should be reserved for cases in which obstruction is suspected. Management should be phenotype-directed, including pathogen-guided antimicrobial therapy when infection is documented, multimodal anti-inflammatory and analgesic strategies integrated with sexual medicine care for CP/CPPS, and time-limited antioxidant supplementation when oxidative imbalance is demonstrated, followed by reassessment and timely escalation to assisted reproductive technologies when indicated.

## Figures and Tables

**Table 1:** National Institutes of Health (NIH) prostatitis categories and fertility-relevant characteristics.

NIH Category	Typical Clinical Features	Pathogen Detection	Fertility-Relevant Semen Findings	Key Evaluation Points
I (Acute bacterial)	Acute febrile illness; pelvic/perineal pain; dysuria.	Usually positive urine or prostatic cultures (typical uropathogens).	Semen testing often deferred; transient impairment due to fever/inflammation; elevated leukocytes/viscosity if assessed.	Treat infection promptly; re-evaluate semen after recovery.
II (Chronic bacterial)	Recurrent urinary tract infection symptoms; relapsing course.	Persistent or recurrent uropathogen in expressed prostatic secretions or semen.	Elevated leukocytes or bacteria in semen; increased oxidative stress; delayed liquefaction; reduced motility.	Quantitative culture and susceptibility; consider biofilm; reassess after therapy.
IIIA (Inflammatory CP/CPPS)	Chronic pelvic pain (>3 months) with inflammatory findings.	Standard cultures often negative; possible dysbiosis or occult pathogens.	Elevated leukocytes and cytokines (e.g., IL-8); oxidative stress; increased sperm DNA fragmentation.	Inflammatory and oxidative biomarkers; targeted microbiology (culture +/− NAAT); phenotype-directed multimodal therapy.
IIIB (Non- inflammatory CP/CPPS)	Chronic pelvic pain without inflammatory cells in semen or EPS.	No pathogen detected.	Semen may be near-normal; fertility impact often via ejaculatory dysfunction and reduced intercourse.	Assess pelvic floor and psychosocial and sexual domains; treat pain and sexual dysfunction.
IV (Asymptomatic inflammatory)	No pain symptoms; inflammation discovered on semen, EPS, or biopsy.	Variable (often no pathogen identified).	Elevated leukocytes and/or cytokines; oxidative stress; possible subtle motility changes.	Consider in “idiopathic” infertility with elevated leukocytes; manage if clinically relevant.

Abbreviations: Abbreviations: NIH, National Institutes of Health; CP/CPPS, chronic prostatitis/chronic pelvic pain syndrome; EPS, expressed prostatic secretion; IL-8, interleukin-8; NAAT, nucleic acid amplification test; DNA, deoxyribonucleic acid.

**Table 2:** Suggested investigations for suspected prostatitis-related male infertility.

Test	When to Consider	What It Answers/ Interpretation	Potential Actions	Threshold/ Examples
Semen analysis (WHO) + leukocyte quantification + viscosity/liquefaction/pH	All men during infertility evaluation; especially if pain/urinary symptoms or prior infection.	Flags inflammatory phenotype (≥1 × 10^6^ WBC/mL) and secretory dysfunction (hyperviscosity/delayed liquefaction).	Triggers targeted microbiology and oxidative/biomarker testing; follow response to therapy.	Leukocytospermia: ≥1.0 × 10^6^ WBC/mL
Seminal cytokines (IL-8 +/− panel)	Suspected accessory-gland inflammation; equivocal leukocytes; persistent symptoms.	Supports prostate-derived inflammation and helps risk-stratify motility impairment.	Strengthens indication for microbiology, oxidative stress assessment, and anti-inflammatory strategy.	IL-8: above lab reference (no universal cutoff)
Oxidative stress testing (ROS assay or oxidation–reduction potential)	Leukocytospermia, infection, CP/CPPS, or unexplained asthenozoospermia.	Quantifies redox imbalance that can damage membranes and DNA.	Address upstream inflammation/infection; consider time-limited antioxidant therapy and lifestyle changes.	ORP: mV/10^6^ sperm/mL; abnormal ~>1.34–1.36 (device/lab-specific)
Sperm DNA fragmentation testing (TUNEL/SCSA/Comet)	Recurrent pregnancy loss, repeated IVF failure, unexplained infertility, or high oxidative stress.	Measures downstream chromatin injury linked to miscarriage and ART outcomes.	Counseling on prognosis; consider advanced sperm selection or testicular sperm in selected high-fragmentation cases.	Assay-/lab-specific abnormal cutoff (report %)
Quantitative semen culture + susceptibility	Inflammatory semen phenotype; symptomatic men; prior prostatitis/UTI history.	Identifies uropathogens and guides targeted antibiotics.	Culture-guided therapy; avoid empiric repeat courses when cultures are negative.	Bacteriospermia: ~≥10^3^ CFU/mL
NAAT for STIs (e.g., *Chlamydia trachomatis*, *Neisseria gonorrhoeae*, *Mycoplasma genitalium*)	Risk factors, persistent inflammation with negative culture, or partner concerns.	Detects fastidious/intracellular pathogens missed by culture.	Treat according to guidance; coordinate partner management to prevent reinfection.	Order NAAT when: STI risk factors; persistent inflammation with negative culture; partner concerns
Symptom scoring/phenotyping (NIH-CPSI +/− UPOINT)	Men with pelvic pain, sexual dysfunction, or CP/CPPS phenotype.	Defines symptom burden and domains driving symptoms/fertility impact.	Guides multimodal therapy and response tracking.	NIH-CPSI: mild ≤ 14; moderate 15–29; severe ≥ 30 (example)
Imaging (transrectal ultrasound +/− MRI)	Very low volume, acidic semen, absent fructose, suspected obstruction/cysts/calcifications.	Assesses ejaculatory duct obstruction and structural sequelae of inflammation.	Select patients for vesiculoscopy/TURED or other corrective interventions.	Suggest obstruction: very low volume; pH < 7.2; fructose absent

Abbreviations: WHO, World Health Organization; WBC, white blood cells; IL-8, interleukin-8; ROS, reactive oxygen species; DNA, deoxyribonucleic acid; ORP, oxidation–reduction potential; TUNEL, terminal deoxynucleotidyl transferase dUTP nick end labeling; SCSA, sperm chromatin structure assay; IVF, in vitro fertilization; ART, assisted reproductive technology; CFU, colony-forming unit; NAAT, nucleic acid amplification test; STI, sexually transmitted infection; UTI, urinary tract infection; NIH-CPSI, National Institutes of Health Chronic Prostatitis Symptom Index; UPOINT, urinary, psychosocial, organ-specific, infection, neurologic/systemic, tenderness; MRI, magnetic resonance imaging; TURED, transurethral resection of the ejaculatory ducts.

## Data Availability

No new data were created or analyzed in this study. Data sharing is not applicable to this article.
